# Physiological recordings: Basic concepts and implementation during functional magnetic resonance imaging

**DOI:** 10.1016/j.neuroimage.2009.05.033

**Published:** 2009-09

**Authors:** Marcus A. Gray, Ludovico Minati, Neil A. Harrison, Peter J. Gianaros, Vitaly Napadow, Hugo D. Critchley

**Affiliations:** a Clinical Imaging Sciences Centre and Department of Psychiatry, Brighton and Sussex Medical School, University of Sussex, Falmer Campus, UK; b Department of Psychiatry, Center for Neural basis of Cognition, University of Pittsburgh, USA; c Athinoula A Martinos Center for Biomedical Imaging, Department of Radiology, Massachusetts General Hospital, Harvard Medical School, USA

## Abstract

Combining human functional neuroimaging with other forms of psychophysiological measurement, including autonomic monitoring, provides an empirical basis for understanding brain–body interactions. This approach can be applied to characterize unwanted physiological noise, examine the neural control and representation of bodily processes relevant to health and morbidity, and index covert expression of affective and cognitive processes to enhance the interpretation of task-evoked regional brain activity. In recent years, human neuroimaging has been dominated by functional magnetic resonance imaging (fMRI) studies. The spatiotemporal information of fMRI regarding central neural activity is valuably complemented by parallel physiological monitoring, yet such studies still remain in the minority. This review article highlights fMRI studies that employed cardiac, vascular, respiratory, electrodermal, gastrointestinal and pupillary psychophysiological indices to address specific questions regarding interaction between brain and bodily state in the context of experience, cognition, emotion and behaviour. Physiological monitoring within the fMRI environment presents specific technical issues, most importantly related to safety. Mechanical and electrical hazards may present dangers to scanned subjects, operator and/or equipment. Furthermore, physiological monitoring may interfere with the quality of neuroimaging data, or itself be compromised by artefacts induced by the operation of the scanner. We review the sources of these potential problems and the current approaches and advice to enable the combination of fMRI and physiological monitoring in a safe and effective manner.

## Introduction

Modern psychophysiology in many respects began in 1898 with John Langley's definition of the autonomic nervous system (ANS) as comprised of the anatomically distinct sympathetic (thoracolumbar nervous system), parasympathetic (craniosacral nervous system) and enteric nervous systems (Langley, 1898; Todman, 2008). Building on this conceptual framework, Walter Cannon in the 1920's outlined how different emotional states may be represented within the brain and expressed in differing patterns of activity within the sympathetic and parasympathetic nervous systems (Cannon, 1927, 1929). Cannon and Hess both emphasized the synchronized and unitary action of the sympathetic nervous system in fright, flight and fight responses, and contrasted these with opposing parasympathetic function, aiding recuperation, conservation of energy and recovery after stress (Cannon, 1939; Hess, 1948). Today it is recognized that activity within the sympathetic and parasympathetic nervous systems are not unitary all or none functions, instead each branch of the autonomic nervous system can independently regulate individual end organs producing complex patterning of autonomic reactions (Jänig, 2006; Paton et al., 2005). There is longstanding recognition of interactions between mental and internal bodily processes, even predating Langley's anatomic description of the autonomic nervous system (ANS) (Langley, 1898). This allows various bodily systems to be kept within narrow homeostatic bounds, while allowing for a range of different meta-stable physiological states necessary to deal effectively with changing cognitive and environmental demands (i.e., allostasis McEwen, 1998). As a consequence, individual psychophysiological measures provide specific information on how sympathetic and parasympathetic activity regulate specific organ systems including the heart, respiratory, circulatory and intestinal systems, eyes, skeletal muscles, skin and viscera.

Despite a growing understanding of how brainstem centers support homoeostatic control, the role of higher regions in supporting cognitive, affective and behavioural influences on autoregulatory processes is less well understood. Over the past 20 years neuroimaging techniques including positron emission tomography (PET) and functional magnetic resonance imaging (fMRI) have substantially advanced the investigation of in vivo cerebral function. Considering simultaneous physiology and neuroimaging data informs understanding of brain–body interaction at multiple levels, with implications for emotional/cognitive neuroscience and psychosomatic medicine. This integrative approach contrasts with the treatment of bodily responses only as confounding sources of noise. Some physiological measures, e.g. the sympathetic skin response (SSR), are relatively commonly used in neuroimaging as ‘independent’ objective indicators of emotional or learning processes (e.g., fear conditioning). Combined electroencephalography (EEG)/fMRI experiments are also increasingly common, motivated by an interest in relationships between different measures of brain activity and facilitated by technical developments and availability of commercial MR-compatible EEG systems. Combining neuroimaging with physiological monitoring permits a more comprehensive neuroscientific account, yet the approach is still in its infancy. While changes in physiological state raise certain difficulties for hemodynamically derived measures of neural function (i.e., BOLD signal in fMRI), the fundamental integration of activity within the central and peripheral nervous system also offers many avenues for detailed examination of brain systems.

This review is an introduction to the simultaneous recording of functional neuroimaging and psychophysiological measures. It specifically aims to 1) introduce a number of physiological measures which, when considered with neuroimaging data, may enrich investigations of body brain interactions within psychosomatic research, and 2) highlight key technical considerations and safety guidelines for acquiring physiological data in the scanning environment. Broader considerations which are not touched in the current review include the conceptualization and interpretation of physiological datasets (e.g., Cacioppo and Tassinary, 1990; O'Connor, 1990; Berntson et al., 1991), integration of experimental design (e.g., Josephs and Henson, 1999; Petersson et al., 1999) with psychophysiological measures, models of physiological noise in MRI signals (e.g., Krüger et al., 2001; Triantafyllou et al., 2005, 2006), physiologically induced autocorrelations in fMRI data analysis (e.g., Casanova et al., 2008; Gautama and Van Hulle, 2004; Bullmore et al., 2001; Worsley, 2005), and Bayesian methods allowing spatial variation in autoregressive models of noise in fMRI data (e.g., Penny et al., 2005; Groves et al., 2009).

### Central nervous system and autonomic measures

Psychophysiology relates individual mental functions to physiological signatures, as exemplified in recent years by functional brain imaging. Historically, peripheral autonomic measures dominated psychophysiology research with cognitive and emotional processes expressed as measurable, but often independent, functional changes in heart, blood vessels, lungs, gastrointestinal tract, eye, skin and muscles. Autonomic outflow is orchestrated in a feedback-dependent hierarchy from local end-organ or spinal reflexes, proximate autonomic nuclei within medulla, pons and lateral hypothalamus, up to higher subcortical and cortical brain regions, including amygdala, insula, orbital and medial prefrontal, cingulate and even primary motor cortex, which couple the regulation of bodily state to motivational behaviours (Benarroch, 1997; Craig, 2002). Here, neuroimaging has greatly enhanced knowledge regarding whole-brain neural mechanisms for efferent modulation and afferent representation of internal bodily state. Below we briefly discuss specific psychophysiological measures within a neuroimaging context.

#### Heart

Sympathetic and parasympathetic (vagus) nerves support the complex neural control of the heart: heart rate, dromotropy and inotropy (rapidity and force of ventricular contractility) are governed by innervation of pacemakers (sinoatrial, SA and atrioventricular, AV, nodes) and additional direct autonomic innervation of the ventricular myocardium. Heart rate and contractility are also modified by humoral agents in addition to local, spinal and bulbospinal chemo, baro and nociceptive reflexes (Weaver et al., 1979; Malliani, 1982; Spyer, 1990; Dampney et al., 2003). Medullary parasympathetic (vagus nerve) nuclei couple cardiac function to blood pressure and respiration: the nucleus of the solitary tract (NTS) regulates blood pressure through baroreceptor reflexes while respiratory medullary neurons mediate respiratory sinus arrhythmia (Jänig, 2006). Baroreceptor and respiratory reflexes also selectively modulate sympathetic outflow to other organs (Dampney et al., 2003). Ultimately, at rest and during physiological and psychological challenge, cardiac function reflects the balance and interacting temporal dynamics of sympathetic and parasympathetic drive (Nalivaiko et al., 2003; Paton et al., 2005).

Within an MRI environment, heart rate provides the most accessible measure of cardiac function as measured through electrocardiography (ECG) or pulse-oximetry. A range of interesting psychophysiological measures can be derived, including overall heart rate, orienting and anticipatory bradycardia, orienting or effort-related tachycardia and beat-to-beat variability in heart rate. Here the usually dominant source of heart rate variability (HRV) tracks respiration and is parasympathetically-mediated. If interbeat intervals are subjected to frequency analysis, parasympathetic influence is most apparent in the high-frequency component of heart rate variability (HF-HRV), while sympathetic influences dominate at lower temporal frequencies (LF-HRV). Increased HF-HRV is related to wellbeing and positive emotions, its withdrawal reflecting stress, low mood and increased cardiovascular medical risk. In fMRI experiments, Matthews et al. (2004) related HF-HRV to ventral ACC activity and O'Connor et al. (2007) demonstrated the relationship between HF-HRV and posterior cingulate activity in people experiencing grief (and diminished HF-HRV).

Perhaps the simplest approach to study spectral dynamics of the heart rate is to apply the Fourier transform; this, however, embeds assumptions of stationarity over a long time window (minutes), which can be an order of magnitude longer than the temporal resolution of the fMRI signal (Napadow et al., 2008). A possible solution to this problem is the use of digital filters to extract high- and low-frequency components (Critchley et al., 2003). Alternatives include the pseudo Wigner–Ville transform and wavelet-based approaches; while offering an improvement in terms of rapidity of response with respect to the Fourier transform, these algorithms still involve a sliding temporal window, which introduces issues of temporal causality as future as well as past data values are taken into account for a given latency (Napadow et al., 2008). The issues of temporal resolution and causality can be addressed by using specific algorithms based on point process methods (Napadow et al., 2008).

**Fig. 1:**
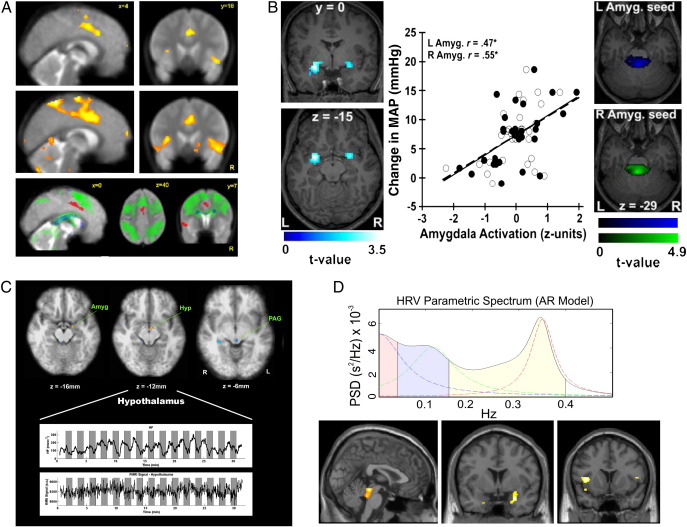
(A) Brain activity associated with sympathetic cardiac control: significant changes correlating with increasing power derived from frequency analysis of interbeat interval (top row orthogonalized low-frequency, sympathetic activity, middle row orthogonalized high-frequency, parasympathetic activity). Segregation of activity relating to effortful cognitive (green) and motor (blue) task performance and activity related to increasing low-frequency power (red) is demonstrated in the bottom row. Reproduced from Critchley et al. (2003) by permission of the copyright holder, Guarantors of Brain. (B) Brain activity associated with mean arterial pressure changes during a stressor task: greater changes in arterial pressure are associated with amygdala activation (left column), and a significant correlation between pressure reactivity and BOLD response is confirmed by a region-of-interest analysis (middle and right column). Arterial pressure changes were also linked to the functional connectivity expressed between the amygdala and pre-autonomic pons (right column). Reproduced from Gianaros et al. (2008) by permission of the copyright holder, Society for Neuroscience.(C). HF-HRV indexed with a point process adaptive-filter algorithm co-varies with functional activity within the amygdala, hypothalamus and periaqueductal gray. (D top) Brain activity associated with heart rate variability during a synchronous/delayed shock stimulation task: power spectra density analysis reveals very low-, low- and high-frequency heart rate variability components. (D bottom) Across subjects, greater high-frequency heart rate variability after delayed shocks correlates with increased BOLD response in the right amygdala and in periaqueductal gray. Panel (D) reproduced from Gray et al. (2009) by permission of the copyright holder, Society for Neuroscience.

Critchley et al. (2003), mapped LF-HRV power changes to dorsal anterior cingulate cortex (dACC) activity (Fig. 1A), and more recently Napadow et al. (2008) indexed dynamic HF-HRV responses to isometric exercise and identified coupling with insular, amygdala and periaqueductal activity (Fig. 1C). Recently, Gray et al. (2009) implicated these same regions in mediating dynamic interactions between HF-HRV and evoked blood pressure responses to somatosensory stimuli (Fig. 1D).

#### Circulation

Autonomic innervation of blood vessels and circulating humoral agents, notably adrenaline and noradrenaline, change peripheral vascular resistance through vasoconstriction and vasodilation. Sympathetic vasoconstriction is regulated in broad somatotomes and selectively reduces local blood flow and volume. One class of sympathetic nerves regulates blood flow to muscles (Jänig, 2006). Muscle sympathetic nerves (MSN) are spontaneously active, but fire in a phasic manner due to potent inhibition during each heartbeat by baroreceptor activation. MSNs are also sensitive to low level visceral, chemoreceptor and respiratory challenges and to central changes that accompany cognition, affect and nociception.

Within an fMRI context, Gianaros et al. (2008) automated blood pressure monitoring to show coupling of amygdala activity with blood pressure changes elicited by mental stress. Enhanced blood pressure reactivity was also reflected in greater functional connectivity between amygdala, perigenual cingulate and pons autonomic regions (Fig. 1B). This extended previous observations linking stress-induced blood pressure change to cingulate cortex, insula and prefrontal activation (Gianaros et al., 2007). Further, Gray et al. (2009) used continuous *non-invasive* blood pressure monitoring to show that sensory-evoked blood pressure changes are gated by both baroreceptor discharge and HF-HRV tone through insula, amygdala and brainstem engagement (Fig. 1D). Functional neural activity assessed by fMRI in central autonomic control areas during other pressor manipulations, including Valsalva maneuvers, cold pressor challenges, and baroreceptor unloading induced by lower body negative pressure, has also been investigated (e.g., Harper et al., 2000; Henderson et al., 2002; Kimmerly et al., 2005; King et al., 1999; Wong et al., 2007). In many of these particular investigations, researchers took the approach of measuring electrocardiographic parameters, muscle sympathetic nerve activity changes, and hemodynamic adjustments outside of the MRI scanning environment (e.g., in a laboratory) and linking these measurements with fMRI patterns of activation to avoid salient safety, technical, and practical challenges detailed herein. Hence, if it is unfeasible for readers of the present review to monitor peripheral physiology in the MRI scanning environment, then the laboratory-based assessments illustrated in the aforementioned studies could be considered.

#### Respiration

In the metabolic control of breathing, chemoreceptive reflexes drive spinal respiratory motor neurons which are also influenced by respiratory pacemaker nuclei within ventrolateral medulla, including the Bötzinger complex and rostral respiratory group (Fortuna et al. 2008). Inspiration and expiration are regulated semi-independently via descending pathways in ventrolateral and ventral spinal columns (Homma and Masaoka, 2008). Respiratory rhythms reflect mutual inhibition between these systems at the level of medullary premotor neurons (von Euler, 1983). Consciously-influenced breathing engages a parallel system of primary motor cortical activation and the recruitment of intercostal muscles and diaphragm (Gandevia and Rothwell, 1987; Gandevia and Plassman, 1988). Importantly, respiratory patterns also strongly respond to emotional and cognitive activity and are enmeshed with cardiac responses. Respiratory motor function is most easily examined within the MRI environment via respiratory bands, thermistors or differential pressure transducers, while the respiratory control network may be examined with respiratory gas analysers and/or controlled ventilation. The global effect of arterial CO_2_ levels on cerebral arteriolar flow is particularly relevant to neuroimaging approaches (including fMRI) that rely on haemodynamic responses to neural activity (Wise et al., 2004). This represents a significant potential confound in studies affecting respiration.

Evans et al. (2002) identified limbic and paralimbic activity during subjective experience of ‘air hunger’, while they controlled for respiratory tidal volume and clamped end-tidal CO_2_ and O_2_ at constant levels. Likewise, Macey et al. (2005) studied patients with congenital central hypoventilation syndrome, identifying cerebellar, thalamic and limbic activation in response to induced hypoxia and hypercapnia in a nitrogen-breathing challenge while controlling for global blood-oxygen level dependent (BOLD) signal changes. In a brainstem-optimized fMRI study, Pattinson et al. (2009) used a CO_2_ respiratory challenge to highlight functional subdivisions of respiratory control centered around the thalamus. They controlled for cerebral vasodilation tracking baseline arterial CO_2_ fluctuations. This paper provides an excellent example of how sophisticated fMRI techniques can be combined with autonomic physiological challenges and careful attention to concomitant physiological responses.

#### Skin and sweat

Skin sympathetic nerves regulate blood vessels, sweat glands and piloerection. There is a topographic distribution of functional activity in these nerves across the body surface (skin of the face, trunk and limbs, hairy or hairless skin) with distinct contributions to thermoregulation and emotional communication (Darwin, 1898; Ekman et al., 1983). Cutaneous vasoconstrictor nerve activity is only weakly regulated by baroreceptors, yet responds strongly to cooling and emotional challenge. Sympathetic nerves also regulate eccrine sweat glands in the palms and the soles of the feet, which do not serve a primary thermoregulatory function (Venables, 1991; Kellogg, 2006). Here, there is no complementary parasympathetic innervation and neurotransmission is cholinergic. Thus the activity of the sweat gland nerves, indexed by the sympathetic skin response (SSR), is not directly sensitive to respiration or circulating adrenaline. SSR in various guises has been widely used as a psychophysiological index of attention and emotion, linked to central mesencephalic reticular, hypothalamus and limbic pathways (Venables and Christie 1980; Gibbins, 1997).

**Fig. 2:**
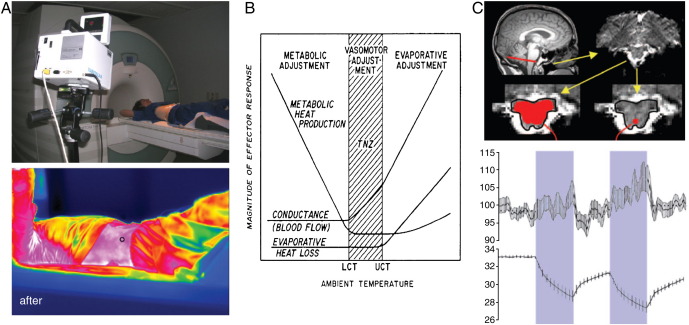
(A top) Infrared camera setup to measure skin temperature in the magnet, and (A bottom) example of a resulting color-map. Reproduced from Boss et al. (2007) by permission of the copyright holder, Wiley-Liss Inc. (B) Thermoregulatory profile of a typical endotherm: below a lower critical threshold (LCT), metabolic heat production gradually increases, whereas above an upper critical threshold (UCT), a steady increase in evaporative heat loss is observed. Reproduced from Adair and Black (2003) by permission of the copyright holder, Wiley-Liss Inc. (C) An FMRI study of skin cooling and rewarming: while correlation with skin temperature was absent for the medulla (left), a significant correlation was found for the raphe nuclei (right) (C top) The time-course of the average BOLD signal in the raphe nuclei (middle) closely follows that of skin temperature (bottom) Panel (C) reproduced from McAllen et al. (2006) by permission of the copyright holder, The National Academy of Sciences of the USA.

There have been few fMRI studies of thermoregulation in humans, however the feasibility of thermal imaging of skin temperature during MRI has been demonstrated (Boss et al., 2007; Fig. 2A). Radio frequency (RF) energy absorbed by the body during fMRI will warm core body temperature over time, with implications for homeostatic thermoregulatory mechanisms (Adair and Black, 2003) (Fig. 2B). McAllen et al. (2006) investigated thermoregulatory mechanisms during fMRI by combined monitoring of skin temperature with a temperature-modifiable garment, identifying activity with the raphé nuclei during cooling and rewarming of the skin (Fig. 2C). More commonly, SSRs are used in neuroimaging studies to index emotional processing, including fear conditioning (Morris et al., 2001; Mobbs et al., 2007). Williams et al. (2001) used SSRs to partition neural responses to fearful faces, finding amygdala activation in association with ‘visceral’ fear during SSR responses and hippocampal activation to declarative fear in the absence of SSR responses. Critchley et al. (2000) also combined SSR and fMRI, to show differential prefrontal cortical activation during generation and afferent representation of SSR events. Similarly, Patterson et al. (2002) demonstrated prefrontal activation during generation of both spontaneous and task-related SSRs.

#### Gastrointestinal responses

Autonomic motor activity within the gut is characterized by travelling peristaltic and motility waves of smooth muscle contraction. These are influenced by pacemaker activity from the interstitial cells of Cajal, autonomic innervation of the myenteric plexus and ultimately by spinal abdominal sympathetic and parasympathetic (vagus nerve) activity (Jänig, 2006). Parasympathetic activity also influences gut blood flow and enzyme secretions (Jänig, 2006). Techniques such as electrogastrography (EGG), measuring electrical waves in the stomach muscle wall, can index gastrointestinal sympathovagal balance and are theoretically usable in the MR environment (Stern et al., 2000; Chang, 2005).

#### Autonomic changes in the eye

Autonomic nervous effects around the eye are most evident in modulation of pupillary size, but also influence eyelid retraction and tear formation. Parasympathetic nerves innervate the iris sphincter muscle (via cranial nerve III cilliary nerve from Edinger–Westphal or accessory oculomotor nucleus) and when active, constrict the pupil (Steinhauer et al., 2004). Conversely, pupil dilation is mediated by parasympathetic withdrawal and by sympathetic innervation of the dilator pupillae muscle from the superior cervical ganglion and long cilliary nerve. Responses to ambient light are initiated by retinal ganglion cells that synapse in olivary pretectal nuclei and then project to the Edinger–Westphal nucleus (Loewenfeld, 1993). Pupillary hippus describes rhythmic but irregular constrictions and dilations of the pupil that occur independently of changes in luminance or eye movements at a frequency of less than 0.04 Hz (McLaren et al., 1992) and are greater in states of drowsiness. Stimuli in all sensory modalities may evoke brief, small amplitude reflex pupillary dilations (Loewenfeld, 1993). Mental or physical effort and changes in emotional state influence pupillary dilation (Kahneman and Beatty, 1966). Within the fMRI environment, eye trackers used to monitor gaze and fixation also provide information on pupillary size. A number of cognitive and emotional studies have used the pupil as an index of emotion or attention, for example Harrison et al. (2008b) examined covariation between one's own and viewed pupil size during viewing of emotional faces. Mismatches in the coherence between observed and perceived pupil size activated extrastriate and corticolimbic regions, supporting the salience of pupillary signals to social and emotional behaviour.

### Implementation of physiological monitoring in the context of neuroimaging with fMRI

The above examples illustrate how physiological measures may inform the study of integrated body and brain responses and provide a more comprehensive understanding of central neural function. Achieving this within neuroimaging requires specific technical considerations that are most obviously highlighted in the context of fMRI.

#### Safety

The combined use of multiple recording devices in the MR scanner presents potential risks to the scanned subject, to staff and to equipment that can be minimized through appropriate adaptation and testing of equipment and the dissemination of knowledge regarding the specific risks and strategies to minimize these. Safety is facilitated by training, equipment certification and adherence to standard operating procedures.

Three types of physical interaction with the MR environment are particularly relevant: magnetically-induced forces and torques, induced voltages and radiofrequency (RF) heating. Each has the potential to generate hazards for the scanned subject and for operators, and may also cause equipment damage (Shellock, 2002; Schaefers and Melzer, 2006; Laufs et al., 2008; Schaefers, 2008). At the time of writing, the key methods for evaluating such risks are those established by the American Society for Testing of Materials (ASTM) International. Broadly, medical devices are classified as MR safe (posing no known danger in any MR environment), MR conditional (posing no danger in specified MR environments and conditions of use) or MR unsafe (ASTM International, F2503-05, 2005). Some passive devices, e.g. plastic- or rubber-made instruments, are intrinsically MR safe, but virtually all active devices are MR conditional, in that they pose no danger only when specified conditions are met. These conditions include: B_0_ magnetic field intensity for devices entering the scanner bore, local field intensity (determined by B_0_ and device position with respect to the magnet) for units remaining outside the bore, constraints on electrode and lead positioning patterns and limitations on the types of RF coils and pulse sequences that can be used (Shellock, 2002; Schaefers and Melzer, 2006; Schaefers 2008). A further distinction is made between devices which are safe from the viewpoint of these three primary interactions but whose *operation* may be affected by, or affect, the MR imaging procedure and devices that can be used in the magnet with no reciprocal interference (often referred to as being MR-compatible) (Shellock, 2002; Schaefers and Melzer, 2006; Schaefers, 2008).

**Fig. 3:**
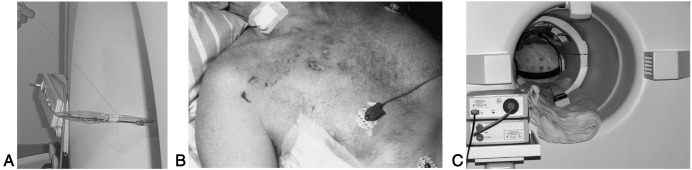
(A) Magnetically-induced force attracting a metallic probe towards the magnet. According to standard safety criteria the deflection angle needs to be less than 45°. Reproduced from Schaefers (2008) by permission of the copyright holder, IEEE. (B) Second-to-third degree burns on the thorax where ECG electrodes were applied, following an incident involving high-voltage induction in long leads. Reproduced from Kugel et al. (2003) by permission of the copyright holder, Springer-Verlag. (C) Reducing lead length, running the conductive wires in parallel near the centre of the magnet and using appropriate padding minimizes risks and improves signal quality. Reproduced from Herrmann and Debener (2008) by permission of the copyright holder, Elsevier.

Hazards related to magnetically-induced force and torque are mechanical in nature, where an object may be displaced and accelerate towards the magnet. Force increases with static magnetic field intensity and spatial gradient at any given point, and with material magnetization and magnetizable mass. A standard safety criterion is that magnetic force should be smaller than gravitational force (Fig. 3A): objects with a high relative content of magnetizable material pose a risk, which diminishes if gravitational force is increased by adding enough amagnetic mass (ASTM international, F2052-06, 2006). Magnetically-induced forces are generally highest at the front and rear of the magnet and lowest at the sides, but they can change abruptly with position, especially on modern, actively shielded systems. Magnetically-induced torque can twist an object and is strongest in the centre of the magnet. It increases with dimensions and material magnetization, and a common criterion is that it should not exceed the maximum gravitational torque (ASTM International, F2213-06, 2006). The effect of dynamic forces and torque due to eddy currents must be also taken into consideration for devices with large, electrically conductive mobile parts (Schaefers, 2008).

During scanning, time-varying magnetic fields are generated by the gradient coils and RF transmitter, which induce voltages across conductive loops and structures. Voltages induced by the imaging gradients increase with slew-rate, and may be large enough to cause nerve stimulation (Schaefer et al., 2000). Voltages induced by coupling with the RF field have been known to cause heating, burns and, in severe cases, even sparks and fire (Knopp et al., 1996; Lemieux et al., 1997; Kugel et al., 2003). Induced voltages can also damage input stages of physiological amplifiers. Official testing criteria are still lacking, but standard measurement methods are proposed (Lemieux et al., 1997; Georgi et al., 2004). Given that the amplitude of the magnetically-induced voltages increases with loop area and number of turns, risks are decreased by ensuring that conductive wires do not form loops and run in parallel close to the centre of the magnet bore (Lemieux et al., 1997). Also, the distance between recording electrodes should be kept to the minimum acceptable value. When multiple, distant recording sites are used (e.g., if recording EMG from both arms), it is important to ensure that each channel is galvanically insulated from the others to prevent the formation of current loops over a large area. The impedance of each electrode connection must be high enough to limit the maximum induced current. This is best achieved using proximal limiting resistors (Lemieux et al., 1997).

Due to conductivity of biological tissues, RF energy induces heating in the patient. The specific absorption rate (SAR) limit of 2 W/kg in normal operating conditions ensures increases in body temperature are within acceptable levels, provided other factors such as room temperature and humidity, bore ventilation and patient physiological conditions are monitored ( International Engineering Consortium, IEC 60601-2-33,2003). Conductive materials (including amagnetic alloys such as electrodes and leads) distort the RF electromagnetic field, potentially leading to local amplification and causing burns. ASTM International has developed a specific standard method of testing medical devices for RF heating, based on a special phantom and fiber optic thermometer (ASTM International F2182-02a, 2002). Unsafe high voltages can be induced through coupling with the electric component of the RF field, even in un-looped cables. This effect increases as lead length approaches half a wavelength (varying between 4.7 and 0.5 m at 1.5 T, depending on the distance of the conductor from the body). Extreme voltages can arise if resonance occurs, but, especially near the transmitter coil, heating and sparking can also occur even in non-resonant circuits. Electrical coupling is thought to have caused severe burns in an incident involving long ECG leads (Fig. 3B) (Kugel et al., 2003). Thus the length of conductive wires should always be minimized (Fig. 3C). Direct connection to recording instruments located outside the magnet room must never be attempted. Although induced voltages are an issue especially for bioelectric recordings, they can also influence the operation of devices which are not electrically connected to the patient — an example is burns caused by incompatible pulse-oximeters (Dempsey and Condon, 2001). Interactions between the RF field and recording devices are often highly non-linear and multifactorial. Exhaustive testing under the specific operating conditions of interest (field strength, coil configuration, pulse sequence and device/lead positioning) is required as extrapolations and assumptions of downwards compatibility are generally not reliable (Lemieux et al., 1997; Helfer et al., 2006; Schaefers and Melzer, 2006; Schaefers, 2008).

In addition to resistors, the use of ferrite sleeves can substantially increase the impedance of connections at RF frequencies, thereby reducing the risk of burns. Only electrodes especially designed for recording in the magnet (typically made of moderately resistive materials) should be used: recording with traditional, metal cup (or similar) electrodes presents a very high risk of causing burns (Krakow et al., 2000). Due to refocusing pulses, SAR is highest for spin–echo sequences, hence recording devices should be disconnected and electrodes removed when performing spin–echo structural imaging. If this is not possible, SAR can be attenuated by reducing the number of slices or increasing repetition time. The use of appropriate padding (e.g., with flame-retardant foam) is highly recommended to ensure that all conductive wires are kept at least 1 cm away from the skin and as distant as possible from the RF coils (Fig. 3C) (Baumann and Noll, 1999; Kettenbach et al., 2006; Herrmann and Debener, 2008).

#### Unwanted physiological interactions and artefacts

Due to its neurovascular origin, the BOLD signal is inevitably sensitive to a range of physiological variables (Friston et al., 2000). While of direct interest to integrative studies of brain–body interaction, changes in physiological state during fMRI task performance may introduce unwanted variance in BOLD data, particularly if the changes are task-related. Cardiac pulsation occurs in arteries and cerebrospinal fluid, especially in brainstem and infratentorial regions (Friese et al., 2004; Dagli et al., 1999). Respiratory changes may be accompanied by bulk movement of the head and thorax which can introduce direct and indirect (modulating local magnetic field strength) variance (Windischberger et al., 2002; Noll and Schneider, 1994). Cardiorespiratory changes are also problematic due to their direct influence on the hemodynamics of the BOLD signal, where CO_2_ concentration modulates the level of vasodilation (Wise et al., 2004). As the cerebrovascular reserve is limited, high levels of CO_2_ can attenuate neurovascular coupling-related changes in the BOLD signal. However, under normal conditions BOLD signal variations due to neural activity and to hypercapnia are largely independent and additive (Corfield et al., 2001). In recent years, considerable effort has gone into the development of methods to estimate and remove from fMRI datasets components correlating with cardiorespiratory function (Brooks et al. 2008; Chang et al. 2009; Birn et al. 2006; Behzadi et al. 2007). This is a considerable challenge for resting-state fMRI studies of default networks: given that cardiorespiratory effects are topographically rather diffuse, they can introduce artefactual correlations among distant regions. This problem is amplified by the fact that permissive filter settings need to be used, in order to preserve sensitivity to slowly fluctuating neural signals (Shmueli et al., 2007).

#### Image artefacts from devices

Recording devices and their accessories can affect MR image quality through distortion of static and imaging gradient magnetic fields, distortion of the RF electromagnetic field and introduction of RF noise. These interactions are especially important when the area to be imaged is close to electrodes, notably with EEG/FMRI, but they can also be significant when recording from other parts of the body (Krakow et al., 2000; Laufs et al., 2008; Mullinger et al., 2008; Dzwonczyk et al., 2009). Although originally introduced for the testing of passive devices, ASTM International F2119-01 remains an important reference for evaluation of image artefacts and IEC 62464-1 also provides standardized means of assessing image quality and degradation (ASTM International F2119-0, 2001; IEC 62464-1, 2007; Schaefers, 2008).

Magnetic field inhomogeneities due to electrodes/devices in or near the magnet bore can cause spatial distortion of the images. Distortion depends primarily on the susceptibility of the device material, and to a smaller extent also on its resistivity due to the effect of eddy currents. Importantly, these artefacts are position- and orientation-dependent, and therefore can change within a session due to subject movement. A distinction is sometimes made between ‘first-kind’ materials, whose susceptibility is small enough for forces and torques not to be problematic and ‘second-kind’ materials that have considerably smaller susceptibility and produce negligible artefacts (Schenck, 1996). Echo-planar imaging sequences are generally most affected due to the small frequency separation between voxels. Here, increasing the bandwidth can reduce distortion, but at the price of increased noise. Signal drop-out may also compromise gradient-echo sequences; this increases with echo time and bandwidth and is frequently more severe than spatial distortion on fMRI datasets (Mullinger et al., 2008). In contrast, spin–echo and fast spin–echo sequences are least affected by magnetic field inhomogeneities.

In practice, distortion of the RF electromagnetic field is usually the dominant cause of image degradation. The effect is strongly dependent on physical dimensions and material resistivity, which determines the intensity of surface screening currents induced in a conductor exposed to an RF field. RF field inhomogeneities introduce artefactual signal variations within the image by reducing the flip angle and the received signal and negatively affect the impedance matching of the coils, decreasing the signal-to-noise ratio (Bartels et al., 2002). These effects are a problem particularly for EEG/FMRI measurements, and their severity increases with both the number of EEG electrodes and the Larmor frequency (Mullinger et al., 2008). Employing high-resistivity electrode materials (such as carbon or ink) and minimizing the amount of conductive gel can help to manage these artefacts (Krakow et al., 2000; Laufs et al., 2008).

The intensity of the RF noise introduced by recording equipment is less position-dependent than the effects of magnetic and RF field inhomogeneities. Depending on its spectral characteristics and on the pulse sequence, RF noise may appear homogeneously distributed, or as spikes or stripes in the image. A common criterion for acceptance is that recording equipment-induced noise should be less than 10% of the receiver-chain noise (e.g., Dzwonczyk et al., 2009). Optical data links should be used wherever possible, as electrical cables passing through the waveguides of the Faraday cage (to be installed only after careful consideration of the induced voltages) present a major source of RF noise. Digital circuitry, electro-mechanical devices (such as pumps and valves used in blood pressure monitors) and switching-mode power supplies may all generate high-frequency components that can be picked up by the receiver and thus should be shielded in separate Faraday cages (Krakow et al., 2000; Laufs et al., 2008; Tang et al., 2008). Decreasing the receiver bandwidth can limit noise contamination but at the price of increased susceptibility and chemical shift artefacts.

#### MRI-induced artefacts in physiological recordings

The static magnetic field can introduce artefacts in bioelectrical recordings even in absence of gradient and RF activity through two routes of interaction: the Hall effect and moving-loop induction. Blood pulsating at high speed in arteries contains charged particles, which get deflected by the Lorenz force and generate a Hall potential across the vessel walls. This is known as the *magnetohydrodynamic* effect and in ECG recordings substantially increase the amplitude of the T-wave, making it difficult to trigger on the R-wave and masking changes characteristic of ischemia (Keltner et al., 1990; Kettenbach et al., 2006; Abi-Abdallah et al., 2007). The use of standard T-wave morphology analysis techniques is therefore severely limited when ECG is recorded in the magnet, calling for further research into algorithms correcting for the magnetohydrodynamic effect.

Together with physical pulsatility of the scalp surface (ballistic effect), the magnetohydrodynamic effect also contributes to generating a heartbeat artefact in EEG recordings, due to blood flow in large arteries of the head. The amplitude of this artefact is often comparable with that of the EEG signal and its morphology resembles that of interictal spikes. Moreover most of its power lies in the frequency range (1–10 Hz) where most EEG activity is found (Allen et al., 1998). Although its periodicity is not perfect, largely because of heart rate variability, an average subtraction method with a sliding window can be used and the residual signal can be removed with principal- or independent-component analysis (Niazy et al., 2005; Grouiller et al., 2007; Laufs et al., 2008).

As a consequence of Faraday's law, a voltage is induced in wire loops moving in a magnetic field. This phenomenon is a prominent source of contamination for bioelectrical recordings in MRI. The resulting artefacts are generally difficult to remove due to varying frequency content and lack of a suitable reference function. To minimize this effect, wires corresponding to differential pairs should be tightly twisted together reducing the loop area and the corresponding electrode impedances should be matched as closely as possible (Goldman et al., 2000). Further, all wires should be secured with tape and sandbags to minimize motion, padded with foam to attenuate gradient vibrations and should run as close as possible to the axis centre of the bore (Baumann and Noll, 1999; Bénar et al., 2003; Laufs et al., 2008). When recording EEG, vacuum head cushions can be used to prevent head movement and reduce the strain associated with lying on the electrodes (Bénar et al., 2003). Moving-loop induction artefacts are a problem for EMG recordings because some degree of movement is inevitable and the artefact is strongly task-correlated. It has been shown, however, that reliable recordings are possible with appropriate electrode and lead positioning (van Duinen et al., 2005). Moving-loop induction is also a potential problem for EGG, given that very low-frequency components are recorded (Stern et al., 2000).

The pump and valves in the cold-head of the MRI scanner can also introduce artefacts into physiological recordings, frequently in the form of spikes appearing approximately every second. If these are present, one could consider (after discussions with the scanner manufacturer) temporarily switching off the cryo-cooler for the duration of the acquisition (Laufs et al., 2008).

**Fig. 4:**
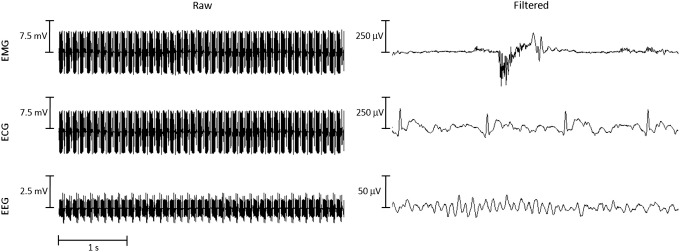
Examples of EMG, ECG and EEG signals before and after artefact removal with the IAR method. The signals were recorded during echo-planar imaging on a 1.5 T whole-body MR scanner. The EMG signal was recorded from electrodes placed bilaterally 2–3 cm apart over the index flexor muscles during brisk extension of the index. The ECG signal was recorded according to the DI lead set from electrodes placed 3–4 cm apart on the chest. The EEG signal was recorded from the O1 electrode, referenced to Fz; ballistocardiogram removal was performed. Please note the different voltage scales for the raw and filtered signal plots. Prior to filtering, the physiological signals are inaccessible; after filtering, the index flexor muscle activity (EMG), R-wave (ECG) and alpha rhythm (EEG) are clearly recognizable. Courtesy of Elisa Visani, Istituto Neurologico “C. Besta”, Milan, Italy.

Imaging gradients introduce artefacts with amplitude that can be more than a thousand times that of the physiological signals of interest (Fig. 4) (Allen et al., 2000; Herrmann and Debener, 2008). Due to the high slew-rate, these artefacts are particularly severe for echo-planar imaging sequences. RF pulses also contaminate the recordings, but the resulting artefact is smaller and considerably shorter (Hamandi et al., 2004; Grouiller et al., 2007). As indicated above, it is important to minimize the length of the leads and the distance between the recording electrodes, in particular with ECG and EMG, and to minimize the impedance of each skin connection (Abächerli et al., 2006; van Duinen et al., 2005). Where feasible, locating the amplification and conversion electronics close to the recording electrodes is advisable (e.g., Lagopoulos et al., 2005).

The amplitude of imaging artefacts usually remains large even under optimal recording conditions necessitating software correction. The first step towards successful artefact removal is ensuring that no information is lost during acquisition. To this end, one needs to choose an input range large enough to avoid clipping, usually a much larger one than those commonly used outside the scanner; for example, ± 15 mV is a common choice for EEG, and is about a hundred times larger than typical ranges for recordings in the EEG lab. To preserve acceptable resolution, a converter with least 16 bits is needed. Imaging artefacts include components at frequencies which are much higher than those found in physiological signals. To enable modelling and removal of these artefacts large pass bands and high sampling rates are necessary: for example 1000 Hz low-pass filtering with 5000 samples/s acquisition is a common choice for EEG in the scanner, compared to around 30 Hz and 500 samples/s for lab-based acquisitions (Allen et al., 2000; Hamandi et al., 2004; Grouiller et al., 2007). Hardware synchronization between the clock of the pulse sequence controller and that of the digitizers improves artefact modelling; if unavailable, a sampling rate of at least 4000 samples/s should be used for EEG to limit the sensitivity to jittering (Mandelkow et al., 2006). Similar considerations also apply to ECG and EMG recordings (Abächerli et al., 2006; van Duinen et al., 2005).

Despite their huge amplitude, imaging artefacts are not difficult to remove thanks to their predictable periodicity. As reviewed by Grouiller et al. (2007) and Ritter et al. (2007), several methods have been proposed, including image artefact reduction (IAR, Allen et al., 2000), fMRI artefact slice template removal (FASTR, Niazy et al., 2005), independent-component analysis (ICA, Jung et al., 2000) and filtering in the frequency domain (Hoffmann et al., 2000). The first step in the IAR method is generation of an average artefact model using a sliding window approach, followed by its subtraction. Adaptive noise cancellation is subsequently performed, iteratively adjusting the filter parameters to minimize correlation between the gradient artefact and the filtered EEG (Allen et al., 2000). In the FASTR method, filtering using the average artefact model is followed by principal-component analysis (PCA); imaging artefacts are typically captured in the first 2–4 components, being much larger than and uncorrelated to the neural signals. Adaptive noise cancellation is subsequently performed (Niazy et al., 2005). Blind source separation by means of ICA can also be used, selecting and removing the components which significantly correlate with an artefact template (Jung et al., 2000). An alternative is filtering in the frequency domain, which is particularly viable when the pulse sequence is optimized to avoid frequency overlap between the artefact and neural activity (Hoffmann et al., 2000). When tested on experimental alpha-rhythm data, IAR and FASTR performed better than ICA and frequency-domain filtering. However, frequency-domain filtering and IAR are preferable to FASTR for spike detection, due to the difficulty in separating spikes from gradient artefacts when using PCA or ICA (Grouiller et al., 2007). It has also been demonstrated that the available implementations of the IAR and FASTR algorithms perform differently in terms of artefact suppression and signal preservation in different frequency bands, highlighting the importance of the selection of the artefact reduction algorithm (Ritter et al., 2007).

IAR and frequency-domain filtering are also applicable to other physiological signals, such as ECG and EMG (Fig. 4) (van Duinen et al., 2005). ECG presents additional challenges when it is used for prospective triggering due to the need for real-time processing. Adaptive noise cancellation, based on a digital finite impulse response filter, has proved successful (Odille et al., 2007).

## Summary

Recording physiological signals simultaneously with fMRI can provide direct information about brain–body interaction and index cognition, affect and covert behaviour. Integration of subject-monitoring approaches with functional brain imaging enables more comprehensive neuroscientific understanding, drawing from a considerable body of psychophysiological literature concerning measures including cardiovascular, respiratory, electrodermal and pupillary expressions of central neural processes. The number of research groups actively pursuing psychophysiological neuroimaging is presently small considering the growth in functional neuroimaging research. Nevertheless, once technical issues are addressed, the results can be highly informative. Accompanying the development of these methods are analytic approaches including linear and non-linear dynamic causal modelling and variants of structural equation modelling, that have gained widespread acceptance and offer new opportunities to explore the interactions between brain activity and body physiology (Penny et al., 2004; Marreiros et al., 2008; Stephan et al., 2008). Integrating peripheral psychophysiological methods with neuroimaging does pose significant technical and methodological challenges, but can usefully inform mechanistic and clinical neuroscientific research.
