# Supplementary material for: Physiological recordings: Basic concepts and implementation during functional magnetic resonance imaging
Source: Neuroimage. 2009 Sep;47(3-8):1105–15. doi: 10.1016/j.neuroimage.2009.05.033 (PMC2741582; doi:10.1016/j.neuroimage.2009.05.033)
Supplement: Supplementary material [file mmc1.doc]

**Figure Reproductions Permissions**

| Figure | Original Author | Authors Permission | Original Journal | Publisher | Publishers Permission |
| --- | --- | --- | --- | --- | --- |
| **1** A | Critchley 2003 | Yes | Brain | Guarantors of Brain | Yes |
| B | Gianaros 2008 | Yes | Journal of Neuroscience | Society for Neuroscience | Waiting for invoice |
| C | Napadow 2008 | Yes | Unpublished | ---- | ---- |
| D | Gray 2009 | Yes | Journal of Neuroscience | Society for Neuroscience | Waiting for invoice |
|  |  |  |  |  |  |
| **2** A | Boss 2007 | Yes | [J Magn Reson Imaging.](javascript:AL_get(this, 'jour', 'J Magn Reson Imaging.');) | Wiley-Liss | Yes |
| B | Adair & Black 2003 | Yes | Bioelectromagnetics Supplement | Wiley-Liss | Yes |
| C | McAllen 2006 | Yes | PNAS | National Academy of Sciences of the USA | Yes |
|  |  |  |  |  |  |
| **3** A | Schaefers 2008 | Yes | IEEE ENGINEERING IN MEDICINE AND BIOLOGY MAGAZINE | IEEE | Yes |
| B | Kugel et al. 2003 | Yes | Eur Radiol | Springer-Verlag | Yes |
| C | Herrmann 2008 | Yes | International Journal of Psychophysiology | Elsevier | Yes |
|  |  |  |  |  |  |
| **4** | Unpublished | Yes | Unpublished | ---- | ---- |
|  |  |  |  |  |  |

Figure 1a – from Critchley (2003).

Publishers Permission

This is a License Agreement between University of Sussex ("You") and Oxford University Press ("Oxford University Press"). The license consists of your order details, the terms and conditions provided by Oxford University Press, and the payment terms and conditions.

Get the printable license.

License Number
       

2136950580023

License date
       

Feb 27, 2009

Licensed content publisher
       

Oxford University Press

Licensed content publication
       

Brain

Licensed content title
       

Human cingulate cortex and autonomic control: converging neuroimaging and clinical evidence

Licensed content author
       

Hugo D. Critchley, et. al.

Licensed content date
       

Jun 23 2003 1:37AM

Type of Use
       

Journal/Magazine

Intended use
       

Commercial

Portion of the article
       

Figures / Tables

Number of figures/tables
       

1

Circulation
       

50000

Periodical name
       

NeuroImage

Title of your work
       

Physiological recordings: basic concepts and implementation in the FMRI scanner (provisional)

Publisher of your work
       

Elsevier

Expected publication date
       

Jun 2009

Billing type
       

Invoice

Company
       

University of Sussex

Billing address
       

Trafford Centre


       


       

Brighton, other BN1 9PH


       

United Kingdom

Customer reference info
       

Permissions cost
       

0.00 USD

Value added tax
       

0.00 USD

Total
       

0.00 USD


Ludovico Minati, MSc
Research fellow
Psychiatry department, Brighton and Sussex Medical School
Trafford Centre
University of Sussex
Falmer
Brighton
BN1 9PH
United Kingdom
+44 784 960 3287
[L.Minati@bsms.ac.uk](mailto:L.Minati@bsms.ac.uk)

Figure 1b – from Gianaros 2008

Publishers Permission Requested – awaiting invoice for payment.

Figure 1d – from Gray 2009

Publishers Permission Requested – awaiting invoice for payment.

**Step 3: Order Confirmation**

**Confirmation Number: 2080319**

**Order Date: 04/28/2009**

If you pay by credit card, your order will be finalized and your card

will be charged within 24 hours. If you pay by invoice, you can

change or cancel your order until the invoice is generated.

Marcus Gray

University of Sussex

m.a.gray@bsms.ac.uk

+44 01273872892

Payment Method: Invoice

**Billing address:**

University of Sussex

CISC, University of Sussex

Falmer Campus

Brighton, East Sussex BN19RR

UNITED KINGDOM

**JOURNAL OF NEUROSCIENCE**

**Order detail ID:**26984942

**Permission Status: Special Order**

**Special Order Update:** Forwarded to

rightsholder

**ISBN/ISSN:** 0270-6474

**Publication year:** 2009

**Publisher:** SOCIETY FOR NEUROSCIENCE

**Rightsholder:** Society for Neuroscience

**Author/Editor:** Gray MA, Rylander K,

Harrison NA, Wallin BG, Critchley HD.

**Permission type:** Republish into a book,

journal, newsletter…

**Requested use:** Journal

**Republication title:** NEUROIMAGE

(PHYSIOLOGICAL RECORDINGS: BASIC

CONCEPTS AND IMPLEMENTATION DURING

FUNCTIONAL MAGNETIC RESONANCE

IMAGING)

**Republishing organization:** ELSEVIER

**Organization status:** For-profit

**Republication date:** 06/01/2009

**Circulation/Distribution:** 10000

**Type of content:** Figure/ diagram/ table

**Description of requested content:**

Following one's heart: cardiac rhythms gate

central initiation of sympathetic reflexes.

**Page range(s):** 29(6), Fig. 4 on p. 1821

**Translating to:** No Translation

**Requested content's publication date:**

02/11/2009

**$TBD**

**(Special Order)**

**JOURNAL OF NEUROSCIENCE**

**Order detail ID:**26984943

**Permission Status: Special Order**

**Special Order Update:** Forwarded to

rightsholder

**ISBN/ISSN:** 0270-6474

**Publication year:** 2008

**Publisher:** SOCIETY FOR NEUROSCIENCE

**Rightsholder:** Society for Neuroscience

**Author/Editor:** Gianaros PJ, Sheu LK,

Matthews KA, Jennings JR, Manuck SB,

Hariri AR.

**Permission type:** Republish into a book,

journal, newsletter…

**Requested use:** Journal

**Republication title:** NEUROIMAGE

(PHYSIOLOGICAL RECORDINGS: BASIC

CONCEPTS AND IMPLEMENTATION DURING

FUNCTIONAL MAGNETIC RESONANCE

IMAGING)

**Republishing organization:** ELSEVIER

**Organization status:** For-profit

**Thank you for your order!** A confirmation for your order will be sent to your account email address. If you have

questions about your order, you can call us at 978-646-2600, M-F between 8:00 AM and 6:00 PM (Eastern), or write

to us at info@copyright.com.

**Payment Information**

**Special Orders**

Copyright Clearance Center https://www.copyright.com/ccc/confirmCartPurchase.do?operation=conf...

1 of 2 28/04/2009 17:25

**Republication date:** 06/01/2009

**Circulation/Distribution:** 10000

**Type of content:** Figure/ diagram/ table

**Description of requested content:**

Individual differences in stressor-evoked

blood pressure reactivity vary with

activation, volume, and functional

connectivity of the amygdala.

**Page range(s):** 28(4), Fig. 2 p. 993 and

fig. 5 p. 995

**Translating to:** No Translation

**Requested content's publication date:**

01/23/2008

**$TBD**

**(Special Order)**

**Order Total: $TBD**

Copyright Clearance Center https://www.copyright.com/ccc/confirmCartPurchase.do?operation=conf...

2 of 2 28/04/2009 17:25

Figure 2a – from Boss (2007)

Publishers Permission

**From:** Goldweber, Paulette - Hoboken [mailto:pgoldweb@wiley.com]
**Sent:** Tue 28/04/2009 15:56
**To:** Minati Ludovico
**Subject:** RE: request for Figs 1 & 3 from Bioelectromagnetics 2007; 26, 5/NeuroImage - Elsevier

Dear Dr. Minati,

This is fine – just use the credit notification that was originally included in our response.

Thanks,

Paulette

Paulette Goldweber| Associate Manager, Permissions| Global Rights - John Wiley & Sons, Inc.

Ph: 201-748-8765 | F: 201-748-6008| [pgoldweb@wiley.com](mailto:pgoldweb@wiley.com)

**From:** L.Minati@bsms.ac.uk [mailto:L.Minati@bsms.ac.uk]
**Sent:** Tuesday, April 28, 2009 10:46 AM
**To:** Goldweber, Paulette - Hoboken
**Subject:** RE: request for Figs 1 & 3 from Bioelectromagnetics 2007; 26, 5/NeuroImage - Elsevier

 Hi, as from our recent conversation, I believe there is a slight mistake in the permission below in that the journal name is wrong - it reads Bioelectromagnetics while it should be J Magn Reson Imaging.

 We seek permission to reproduce Figures 1 (page 1336) and 3 (page 1337) from

 Boss A, Graf H, Berger A, Lauer UA, Wojtczyk H, Claussen CD, Schick F. Tissue warming and regulatory responses induced by radio frequency energy deposition on a whole-body 3-Tesla magnetic resonance imager. J Magn Reson Imaging. 2007 Nov;26(5):1334-9

 in an invited review article, Gray, Minati et al. Physiological Recordings: Basic Concepts and Implementation During Functional Magnetic Resonance Imaging to appear on a special June issue of the journal Neuroimage.

 We apologize but as we did not spot this problem before and the article is due out today, I would be sincerely grateful if we could obtain the above permission.

 My full details are in the signature of this message.

Thank you and regards,

Ludovico Minati

 Ludovico Minati, MSc

Research fellow

Psychiatry department, Brighton and Sussex Medical School

Trafford Centre

University of Sussex

Falmer

Brighton

BN1 9PH

United Kingdom

+44 784 960 3287

[L.Minati@bsms.ac.uk](mailto:L.Minati@bsms.ac.uk)

Figure 2b – from Adair & Black (2003).

Publishers Permission

**From:** Goldweber, Paulette - Hoboken [mailto:pgoldweb@wiley.com]
**Sent:** Tue 28/04/2009 16:20
**To:** Minati Ludovico
**Subject:** RE: request for Figs 1 & 3 from Bioelectromagnetics 2007; 26, 5/NeuroImage - Elsevier

That’s fine as well.

Thanks.

Paulette Goldweber| Associate Manager, Permissions| Global Rights - John Wiley & Sons, Inc.

Ph: 201-748-8765 | F: 201-748-6008| [pgoldweb@wiley.com](mailto:pgoldweb@wiley.com)

**From:** L.Minati@bsms.ac.uk [mailto:L.Minati@bsms.ac.uk]
**Sent:** Tuesday, April 28, 2009 11:10 AM
**To:** Goldweber, Paulette - Hoboken
**Subject:** RE: request for Figs 1 & 3 from Bioelectromagnetics 2007; 26, 5/NeuroImage - Elsevier

I am sorry for additional request - in fact I believe the problem was that 2 permissions requests got merged/confused somewhat.

In addition to what I wrote in the previous email, we also need permission to reproduce Figure 2 (page S26) from

Adair ER, Black DR. Thermoregulatory responses to RF energy absorption.
Bioelectromagnetics. 2003;Suppl 6:S17-38

Again, this will appear in an invited review article, Gray, Minati et al. Physiological Recordings: Basic Concepts and Implementation During Functional Magnetic Resonance Imaging to appear on a special June issue of the journal Neuroimage (Elsevier)

Thank you very much again and regards,

Ludovico Minati

Ludovico Minati, MSc

Research fellow

Psychiatry department, Brighton and Sussex Medical School

Trafford Centre

University of Sussex

Falmer

Brighton

BN1 9PH

United Kingdom

+44 784 960 3287

[L.Minati@bsms.ac.uk](mailto:L.Minati@bsms.ac.uk)

**From:** Goldweber, Paulette - Hoboken [mailto:pgoldweb@wiley.com]
**Sent:** Wed 25/03/2009 15:58
**To:** Minati Ludovico
**Subject:** re: request for Figs 1 & 3 from Bioelectromagnetics 2007; 26, 5/NeuroImage - Elsevier

John Wiley & Sons, Inc. has no objections to your proposed reuse of this material.

Credit to our Work must appear on every copy using the Material as follows: Title, volume number, issue number, year (e.g. Vol. 1, No. 1, 1996), page numbers.  Copyright  (year and owner); and the statement  “Reprinted with permission of John Wiley & Sons, Inc.”

NOTE: No rights are granted to use content that appears in the work with credit to another source.

Paulette Goldweber| Associate Manager, Permissions| Global Rights - John Wiley & Sons, Inc.

Ph: 201-748-8765 | F: 201-748-6008| [pgoldweb@wiley.com](mailto:pgoldweb@wiley.com)

Figure 2c – from McAllen (2009).

Publishers Permission

Dear Dr. Minati,

Permission is granted for your use of the figure as described in your message below.  Please cite the full journal references and "Copyright (copyright year) National Academy of Sciences, U.S.A."

 Best regards,

Kelly Newton for

Diane Sullenberger

Executive Editor

PNAS

**From:** L.Minati@bsms.ac.uk [mailto:L.Minati@bsms.ac.uk]
**Sent:** Friday, February 27, 2009 5:10 AM
**To:** PNAS Permissions
**Subject:** permissions request
**Importance:** High

Dear sirs,

        my co-authors and I are preparing a review article, tentative title ‘Physiological recordings: basic concepts and implementation in the FMRI scanner’, first author ‘Gray MA’, to be submitted for consideration by the journal ‘NeuroImage’, published by Elsevier.

        We seek permission to reproduce Fig. 1 from page 812 of McAllen et al. ‘Human medullary responses to cooling and rewarming the skin: a functional MRI study.’ Proc Natl Acad Sci U S A. 2006; 103(3):809-13

        Credit will be given to the authors, who will be notified in writing, and a copyright notice will appear in your name.        Thanks in advance and kind regards,

Ludovico Minati


Ludovico Minati, MSc
Research fellow
Psychiatry department, Brighton and Sussex Medical School
Trafford Centre
University of Sussex
Falmer
Brighton
BN1 9PH
United Kingdom

Figure 3a – from Schafers (2008).

Publishers Permission

Comments/Response to Case ID: 003A041D

ReplyTo: Copyrights@ieee.org
                                                                                
      From: Jacqueline Hansson            Date: 03/16/2009                      
                                                                         
   Subject: Re: Permissions            Send To: L.Minati@bsms.ac.uk             
            request                                                             
                                                                                
                                            cc:                                 
 Dear Ludovico Minati

This is in response to your letter below, in which you have requested
permission to reprint the described IEEE copyrighted figure.  We are happy
to grant you permission to use the figure in all printed and electronic
formats.

Our only requirements are that you credit the original source (author,
paper, and publication), and that the IEEE copyright line (©2008 IEEE)
appears prominently with the reprinted figure.

Sincerely,

Jacqueline Hansson


©  ©  ©  ©  ©  ©  ©  ©  ©  ©  ©  ©  ©  ©  ©  ©  ©  ©
IEEE Intellectual Property Rights Office
445 Hoes Lane
Piscataway, NJ  08855-1331 USA
+1 732 562 3966 (phone)
+1 732 562 1746 (fax)

IEEE-- Fostering technological innovation
and excellence for the benefit of humanity.
©  ©  ©  ©  ©  ©  ©  ©  ©  ©  ©  ©  ©  ©  ©  ©  ©  ©


Dear sirs,

      my co-authors and I are preparing a review article, tentative title
‘Physiological recordings: basic concepts and implementation in the FMRI
scanner’, to be submitted for consideration by the journal ‘NeuroImage’,
published by Elsevier, and would like to obtain permission to reproduce
Fig. 1 from page 24 of Schaefers G. Testing MR safety and compatibility: an
overview of the methods and current standards. IEEE Eng Med Biol Mag. 2008;
27(3):23-7. Credit will be given to the authors and a copyright notice will
appear in your name.

      Looking forward to your prompt reply, kind regards

Ludovico Minati


Ludovico Minati, MSc
Research fellow
Psychiatry department, Brighton and Sussex Medical School
Trafford Centre
University of Sussex
Falmer
Brighton
BN1 9PH
United Kingdom
+44 784 960 3287
L.Minati@bsms.ac.uk

Figure 3b – from Kugel (2003).

Publishers Permission

SPRINGER LICENSE
TERMS AND CONDITIONS

Feb 26, 2009


This is a License Agreement between University of Sussex ("You") and Springer ("Springer") provided by Copyright Clearance Center ("CCC"). The license consists of your order details, the terms and conditions provided by Springer, and the payment terms and conditions.

All payments must be made in full to CCC. For payment instructions, please see information listed at the bottom of this form.

License Number
       

2136451061640

License date
       

Feb 26, 2009

Licensed content publisher
       

Springer

Licensed content publication
       

European Radiology

Licensed content title
       

Hazardous situation in the MR bore: induction in ECG leads causes fire

Licensed content author
       

Harald Kugel

Licensed content date
       

Apr 1, 2003

Volume number
       

13

Issue number
       

4

Pages
       

690 - 694

Type of Use
       

Journal/Magazine

Author of licensed content
       

No, I am not an author of the article requested.

Author or editor of new article
       

Yes, I am an author or editor of the new article.

Requestor Type
       

Publisher (STM-Signatory)

Portion of the article
       

Figures

Number of figures
       

1

Print run
       

500000

Title of your article
       

Physiological recordings: basic concepts and implementation in the FMRI scanner (provisional)

Publication of your article
       

NeuroImage

Publisher of your article
       

Elsevier

Expected publication date
       

Jun 2009

Estimated size (pages)
       

10

Billing Type
       

Invoice

Company
       

University of Sussex

Billing Address
       

Trafford Centre


       


       

Brighton, other BN1 9PH


       

United Kingdom

Customer reference info
       

Total
       

0.00 USD

Terms and Conditions

Introduction
The publisher for this copyrighted material is Springer Science + Business Media. By clicking "accept" in connection with completing this licensing transaction, you agree that the following terms and conditions apply to this transaction (along with the Billing and Payment terms and conditions established by Copyright Clearance Center, Inc. ("CCC"), at the time that you opened your Rightslink account and that are available at any time at [http://myaccount.copyright.com](http://myaccount.copyright.com/)).

Limited License
Springer Science + Business Media hereby grants to you a non-exclusive license to use this material, for the use as indicated in your inquiry. Licenses are for one-time use only with a maximum distribution equal to the number that you identified in the licensing process.

This License includes use in an electronic form, provided it's password protected, on intranet, or CD-Rom/E-book. For any other electronic use, please contact Springer at [permissions.dordrecht@springer.com](mailto:permissions.dordrecht@springer.com) or [permissions.heidelberg@springer.com](mailto:permissions.heidelberg@springer.com)

Although Springer holds copyright to the material and is entitled to negotiate on rights, this license is only valid, provided permission is also obtained from the author (address is given with the article/chapter) and provided it concerns original material which does not carry references to other sources (if material in question appears with credit to another source, authorization from that source is required as well).

Geographic Rights: Scope
Licenses may be exercised anywhere in the world.

Altering/Modifying Material: Not Permitted
However figures and illustrations may be altered minimally to serve your work. Any other abbreviations, additions, deletions and/or any other alterations shall be made only with prior written authorization of the author(s) and/or Springer Science + Business Media. (Please contact Springer at [permissions.dordrecht@springer.com](mailto:permissions.dordrecht@springer.com) or [permissions.heidelberg@springer.com](mailto:permissions.heidelberg@springer.com))

Reservation of Rights
Springer Science + Business Media reserves all rights not specifically granted in the combination of (i) the license details provided by you and accepted in the course of this licensing transaction, (ii) these terms and conditions and (iii) CCC's Billing and Payment terms and conditions.

License Contingent on Payment
While you may exercise the rights licensed immediately upon issuance of the license at the end of the licensing process for the transaction, provided that you have disclosed complete and accurate details of your proposed use, no license is finally effective unless and until full payment is received from you (either by Springer Science + Business Media or by CCC) as provided in CCC's Billing and Payment terms and conditions. If full payment is not received on a timely basis, then any license preliminarily granted shall be deemed automatically revoked and shall be void as if never granted. Further, in the event that you breach any of these terms and conditions or any of CCC's Billing and Payment terms and conditions, the license is automatically revoked and shall be void as if never granted. Use of materials as described in a revoked license, as well as any use of the materials beyond the scope of an unrevoked license, may constitute copyright infringement and Springer Science + Business Media reserves the right to take any and all action to protect its copyright in the materials.

Copyright Notice:
Please include the following copyright citation referencing the publication in which the material was originally published. Where wording is within brackets, please include verbatim.

"With kind permission from Springer Science+Business Media: <book/journal title, chapter/article title, volume, year of publication, page, name(s) of author(s), figure number(s), and any original (first) copyright notice displayed with material>."

Warranties
Springer Science + Business Media makes no representations or warranties with respect to the licensed material.

Indemnity
You hereby indemnify and agree to hold harmless Springer Science + Business Media and CCC, and their respective officers, directors, employees and agents, from and against any and all claims arising out of your use of the licensed material other than as specifically authorized pursuant to this license.

No Transfer of License
This license is personal to you and may not be sublicensed, assigned, or transferred by you to any other person without Springer Science + Business Media's written permission.

No Amendment Except in Writing
This license may not be amended except in a writing signed by both parties (or, in the case of Springer Science + Business Media, by CCC on Springer Science + Business Media's behalf).

Objection to Contrary Terms
Springer Science + Business Media hereby objects to any terms contained in any purchase order, acknowledgment, check endorsement or other writing prepared by you, which terms are inconsistent with these terms and conditions or CCC's Billing and Payment terms and conditions. These terms and conditions, together with CCC's Billing and Payment terms and conditions (which are incorporated herein), comprise the entire agreement between you and Springer Science + Business Media (and CCC) concerning this licensing transaction. In the event of any conflict between your obligations established by these terms and conditions and those established by CCC's Billing and Payment terms and conditions, these terms and conditions shall control.

Jurisdiction
All disputes that may arise in connection with this present License, or the breach thereof, shall be settled exclusively by the country's law in which the work was originally published.

v1.3

Gratis licenses (referencing $0 in the Total field) are free. Please retain this printable license for your reference. No payment is required.

If you would like to pay for this license now, please remit this license along with your payment made payable to "COPYRIGHT CLEARANCE CENTER" otherwise you will be invoiced within 30 days of the license date. Payment should be in the form of a check or money order referencing your account number and this license number 2136451061640.
If you would prefer to pay for this license by credit card, please go to <http://www.copyright.com/creditcard> to download our credit card payment authorization form.

Make Payment To:
Copyright Clearance Center
Dept 001
P.O. Box 843006
Boston, MA 02284-3006

If you find copyrighted material related to this license will not be used and wish to cancel, please contact us referencing this license number 2136451061640 and noting the reason for cancellation.

Questions? [customercare@copyright.com](mailto:customercare@copyright.com) or +1-877-622-5543 (toll free in the US) or +1-978-646-2777.


Ludovico Minati, MSc
Research fellow
Psychiatry department, Brighton and Sussex Medical School
Trafford Centre
University of Sussex
Falmer
Brighton
BN1 9PH
United Kingdom
+44 784 960 3287
[L.Minati@bsms.ac.uk](mailto:L.Minati@bsms.ac.uk)

Figure 3c – from Herman (2009).

Publishers Permission

TERMS AND CONDITIONS

Mar 24, 2009

This is a License Agreement between University of Sussex ("You") and Elsevier ("Elsevier") provided by Copyright Clearance Center ("CCC"). The license consists of your order details, the terms and conditions provided by Elsevier, and the payment terms and conditions.

**All payments must be made in full to CCC. For payment instructions, please see information listed at the bottom of this form.**

Supplier

Elsevier Limited
The Boulevard,Langford Lane
Kidlington,Oxford,OX5 1GB,UK

Registered Company Number

1982084

Customer name

University of Sussex

Customer address

Trafford Centre

Brighton, other BN1 9PH

License Number

2155470172129

License date

Mar 24, 2009

Licensed content publisher

Elsevier

Licensed content publication

International Journal of Psychophysiology

Licensed content title

Simultaneous recording of EEG and BOLD responses: A historical perspective

Licensed content author

Christoph S. Herrmann and Stefan Debener

Licensed content date

March 2008

Volume number

Issue number

Pages

0

Type of Use

Journal/Magazine

Requestor type

Author of new work

Portion

Figures/table/illustration/abstracts

Portion Quantity

1

Format

Both print and electronic

You are an author of the Elsevier article

No

Are you translating?

No

Order Reference Number

Government agency title

NeuroImage

Title of your report

Physiological recordings: basic concepts and implementation in the FMRI scanner (provisional)

Publisher of your report

Elsevier

Expected publication date

Jun 2009

Estimated size of your report (pages)

10

Elsevier VAT number

GB 494 6272 12

Permissions price

0.00 USD

Value added tax 0.0%

0.00 USD

(0.0 £)

Total

0.00 USD

Terms and Conditions

**INTRODUCTION**

1. The publisher for this copyrighted material is Elsevier.  By clicking "accept" in connection with completing this licensing transaction, you agree that the following terms and conditions apply to this transaction (along with the Billing and Payment terms and conditions established by Copyright Clearance Center, Inc. ("CCC"), at the time that you opened your Rightslink account and that are available at any time at [http://myaccount.copyright.com](http://myaccount.copyright.com/)).

**GENERAL TERMS**

2. Elsevier hereby grants you permission to reproduce the aforementioned material subject to the terms and conditions indicated.

3. Acknowledgement: If any part of the material to be used (for example, figures) has appeared in our publication with credit or acknowledgement to another source, permission must also be sought from that source.  If such permission is not obtained then that material may not be included in your publication/copies. Suitable acknowledgement to the source must be made, either as a footnote or in a reference list at the end of your publication, as follows:

“Reprinted from Publication title, Vol /edition number, Author(s), Title of article / title of chapter, Pages No., Copyright (Year), with permission from Elsevier [OR APPLICABLE SOCIETY COPYRIGHT OWNER].” Also Lancet special credit - “Reprinted from The Lancet, Vol. number, Author(s), Title of article, Pages No., Copyright (Year), with permission from Elsevier.”

4. Reproduction of this material is confined to the purpose and/or media for which permission is hereby given.

5. Altering/Modifying Material: Not Permitted. However figures and illustrations may be altered/adapted minimally to serve your work. Any other abbreviations, additions, deletions and/or any other alterations shall be made only with prior written authorization of Elsevier Ltd. (Please contact Elsevier at permissions@elsevier.com)

6. If the permission fee for the requested use of our material is waived in this instance, please be advised that your future requests for Elsevier materials may attract a fee.

7. Reservation of Rights: Publisher reserves all rights not specifically granted in the combination of (i) the license details provided by you and accepted in the course of this licensing transaction, (ii) these terms and conditions and (iii) CCC's Billing and Payment terms and conditions.

8. License Contingent Upon Payment: While you may exercise the rights licensed immediately upon issuance of the license at the end of the licensing process for the transaction, provided that you have disclosed complete and accurate details of your proposed use, no license is finally effective unless and until full payment is received from you (either by publisher or by CCC) as provided in CCC's Billing and Payment terms and conditions.  If full payment is not received on a timely basis, then any license preliminarily granted shall be deemed automatically revoked and shall be void as if never granted.  Further, in the event that you breach any of these terms and conditions or any of CCC's Billing and Payment terms and conditions, the license is automatically revoked and shall be void as if never granted.  Use of materials as described in a revoked license, as well as any use of the materials beyond the scope of an unrevoked license, may constitute copyright infringement and publisher reserves the right to take any and all action to protect its copyright in the materials.

9. Warranties: Publisher makes no representations or warranties with respect to the licensed material.

10. Indemnity: You hereby indemnify and agree to hold harmless publisher and CCC, and their respective officers, directors, employees and agents, from and against any and all claims arising out of your use of the licensed material other than as specifically authorized pursuant to this license.

11. No Transfer of License: This license is personal to you and may not be sublicensed, assigned, or transferred by you to any other person without publisher's written permission.

12. No Amendment Except in Writing: This license may not be amended except in a writing signed by both parties (or, in the case of publisher, by CCC on publisher's behalf).

13. Objection to Contrary Terms: Publisher hereby objects to any terms contained in any purchase order, acknowledgment, check endorsement or other writing prepared by you, which terms are inconsistent with these terms and conditions or CCC's Billing and Payment terms and conditions.  These terms and conditions, together with CCC's Billing and Payment terms and conditions (which are incorporated herein), comprise the entire agreement between you and publisher (and CCC) concerning this licensing transaction.  In the event of any conflict between your obligations established by these terms and conditions and those established by CCC's Billing and Payment terms and conditions, these terms and conditions shall control.

14. Revocation: Elsevier or Copyright Clearance Center may deny the permissions described in this License at their sole discretion, for any reason or no reason, with a full refund payable to you.  Notice of such denial will be made using the contact information provided by you.  Failure to receive such notice will not alter or invalidate the denial.  In no event will Elsevier or Copyright Clearance Center be responsible or liable for any costs, expenses or damage incurred by you as a result of a denial of your permission request, other than a refund of the amount(s) paid by you to Elsevier and/or Copyright Clearance Center for denied permissions.

**LIMITED LICENSE**

The following terms and conditions apply to specific license types:

15. **Translation**: This permission is granted for non-exclusive world **English** rights only unless your license was granted for translation rights. If you licensed translation rights you may only translate this content into the languages you requested. A professional translator must perform all translations and reproduce the content word for word preserving the integrity of the article. If this license is to re-use 1 or 2 figures then permission is granted for non-exclusive world rights in all languages.

16. **Website**: The following terms and conditions apply to electronic reserve and author websites:
**Electronic reserve**: If licensed material is to be posted to website, the web site is to be password-protected and made available only to bona fide students registered on a relevant course if:
This license was made in connection with a course,
This permission is granted for 1 year only. You may obtain a license for future website posting,
All content posted to the web site must maintain the copyright information line on the bottom of each image,
A hyper-text must be included to the Homepage of the journal from which you are licensing at <http://www.sciencedirect.com/science/journal/xxxxx> or, for books, to the Elsevier homepage at [http://www.elsevier.com](http://www.elsevier.com/),
Central Storage: This license does not include permission for a scanned version of the material to be stored in a central repository such as that provided by Heron/XanEdu.

17. **Author website** for journals with the following additional clauses:

All content posted to the web site must maintain the copyright information line on the bottom of each image, and
The permission granted is limited to the personal version of your paper.  You are not allowed to download and post the published electronic version of your article (whether PDF or HTML, proof or final version), nor may you scan the printed edition to create an electronic version,
A hyper-text must be included to the Homepage of the journal from which you are licensing at <http://www.sciencedirect.com/science/journal/xxxxx>,
Central Storage: This license does not include permission for a scanned version of the material to be stored in a central repository such as that provided by Heron/XanEdu.

18. **Author website** for books with the following additional clauses:
Authors are permitted to place a brief summary of their work online only.
A hyper-text must be included to the Elsevier homepage at [http://www.elsevier.com](http://www.elsevier.com/).
All content posted to the web site must maintain the copyright information line on the bottom of each image
You are not allowed to download and post the published electronic version of your chapter, nor may you scan the printed edition to create an electronic version.
Central Storage: This license does not include permission for a scanned version of the material to be stored in a central repository such as that provided by Heron/XanEdu.

19. **Website** (regular and for author): A hyper-text must be included to the Homepage of the journal from which you are licensing at <http://www.sciencedirect.com/science/journal/xxxxx> or, for books, to the Elsevier homepage at [http://www.elsevier.com](http://www.elsevier.com/).

20. **Thesis/Dissertation**: If your license is for use in a thesis/dissertation your thesis may be submitted to your institution in either print or electronic form. Should your thesis be published commercially, please reapply for permission. These requirements include permission for the Library and Archives of Canada to supply single copies, on demand, of the complete thesis and include permission for UMI to supply single copies, on demand, of the complete thesis. Should your thesis be published commercially, please reapply for permission.

21. **Other conditions:** None

v1.5

**Gratis licenses (referencing $0 in the Total field) are free. Please retain this printable license for your reference. No payment is required.**

**If you would like to pay for this license now, please remit this license along with your payment made payable to "COPYRIGHT CLEARANCE CENTER" otherwise you will be invoiced within 30 days of the license date. Payment should be in the form of a check or money order referencing your account number and this license number 2155470172129.
If you would prefer to pay for this license by credit card, please go to** [**http://www.copyright.com/creditcard**](http://www.copyright.com/creditcard) **to download our credit card payment authorization form.

Make Payment To:
Copyright Clearance Center
Dept 001
P.O. Box 843006
Boston, MA 02284-3006

If you find copyrighted material related to this license will not be used and wish to cancel, please contact us referencing this license number 2155470172129 and noting the reason for cancellation.

Questions?** [**customercare@copyright.com**](mailto:customercare@copyright.com) **or +1-877-622-5543 (toll free in the US) or +1-978-646-2777.**
